# Photoactivated Enzymatic Reaction Network Enables Spatiotemporal Programming of Thiol/Disulfide Redox Systems

**DOI:** 10.1002/anie.202503822

**Published:** 2025-05-05

**Authors:** Aritra Sarkar, Piet J. M. Swinkels, Lea Duttenhofer, Pol Besenius, Andreas Walther

**Affiliations:** ^1^ Department of Chemistry University of Mainz Duesbergweg 10–14 55128 Mainz Germany

**Keywords:** Chemical reaction network, Hydrogels, Spatiotemporal control, Systems chemistry, Thiol‐disulfide system

## Abstract

Implementing photoactivation into chemical reaction networks (CRNs) offers opportunities for on‐demand and remote activation, fuel storage, creating autonomous materials and systems with precise spatial activation. However, examples in this domain remain limited. Here, we introduce a cascaded redox‐based enzymatic reaction network (ERN) capable of photoinitiation and refueling, centered around dissipative aromatic disulfide bond formation. To achieve photoinitiation, we integrate an upstream enzymatic module (EM) into the ERN, where the substrate of the EM can be photouncaged using blue light irradiation. A downstream regeneration system enhances the robustness of the reducing environment for repeated fueling. This cascaded ERN operates in solution, allowing for controlled dissipative disulfide formation while avoiding short‐circuit reactions (SCR) between the oxidative and reductive halves of the reaction cycle. To showcase the utility of this photoactivated ERN, we couple it with aromatic thiol‐terminated star polymers (sPEG‐ArSH) to enable spatiotemporally controlled dissipative hydrogel formation using lithographic masks.

## Graphical abstract

This work introduces a redox‐based enzymatic reaction network that can be photoactivated and recharged multiple times to sustain dissipative disulfide formation. The innovative system integrates an upstream oxidative enzymatic process with a downstream reducing agent‐regenerating enzymatic mechanism. The redox‐based enzymatic reaction network facilitates lithographic programming of sol‐gel transformation in a hydrogel material system.

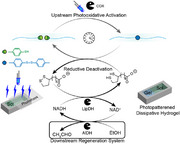

## Introduction

Fuel‐driven chemical reaction networks (CRNs) lie at the heart of biological systems, enabling spatiotemporal and adaptive behaviors.^[^ ^1^, ^2^, ^3^, ^4^, ^5^, ^6^ ^]^ At the molecular level, these CRNs are well‐studied, e.g., in the context of phosphorylation/dephosphorylation cycles.^[^ ^7^, ^8^, ^9^ ^]^ Inspired by these natural processes, a variety of artificial, fuel‐driven CRNs have been developed, aimed at creating autonomous and programmable molecular systems^[^ ^10^, ^11^, ^12^, ^13^, ^14^, ^15^, ^16^, ^17^, ^18^, ^19^, ^20^, ^21^, ^22^, ^23^, ^24^, ^25^, ^26^, ^27^, ^28^, ^29^ ^]^ in the field of systems chemistry.^[^ ^30^, ^31^, ^32^, ^33^, ^34^, ^35^, ^36^, ^37^ ^]^ Developing photoactivation or photofueling of CRNs is important for non‐invasive and precise fuel delivery, capacity for on‐demand fuel storage and release, and spatiotemporal activation of systems.^[^ ^38^, ^39^, ^40^, ^41^, ^42^, ^43^ ^]^ So far, strategies involve coupling of photoswitchable molecules with nanoparticles,^[^ ^44^, ^45^, ^46^, ^47^, ^48^, ^49^ ^]^ use of photoprotecting groups to photouncage ATP in ATP‐fueled, DNA‐based enzymatic reaction networks,^[^ ^50^ ^]^ photouncaging of protected thiol molecules for transient softening of polymer network,^[^ ^51^ ^]^ and use of photoacids to initiate pH‐driven DNA based CRNs.^[^ ^52^ ^]^ Advancing photocontrol over redox‐based CRNs^[^ ^53^, ^54^, ^55^, ^56^ ^]^ could significantly expand their applicability in material systems, yet, examples in this area remain very limited. To date, existing work includes the photocatalytic reduction and oxidation of self‐assembling molecules to initiate CRNs via light.^[^ ^57^ ^]^

Reversible thiol‐disulfide‐based dynamic covalent bonds represent one of the most significant redox‐responsive systems, with applications in areas such as hydrogels,^[^ ^58^ ^]^ self‐assemblies,^[^ ^59^ ^]^ and synthetic self‐replicators.^[^ ^60^, ^61^ ^]^ However, these systems have mainly been studied in the responsive regime, and their integration into fuel‐driven CRNs remains a challenge^[^ ^62^, ^63^, ^64^, ^65^, ^66^ ^]^ due to the direct annihilation of the oxidizing fuel in a reducing environment, leading to short circuit reactions (SCRs). Recently, we presented an enzymatic reaction network (ERN) that integrates thiol‐disulfide chemistry into fuel‐driven, out‐of‐equilibrium CRNs, and we showed how to overcome SCRs.^[^ ^67^ ^]^ Despite these advances, a photoinitiated thiol‐disulfide‐based dissipative redox cycle is yet to be achieved—a key step toward spatiotemporal control over thiol‐disulfide systems. In this work, we expand our previous ERN by introducing dedicated upstream and downstream enzymatic modules (EMs) to introduce photofueling (pERN) upstream and enhance the robustness for multiple fuel cycles with a downstream regeneration system.

**Figure 1:**
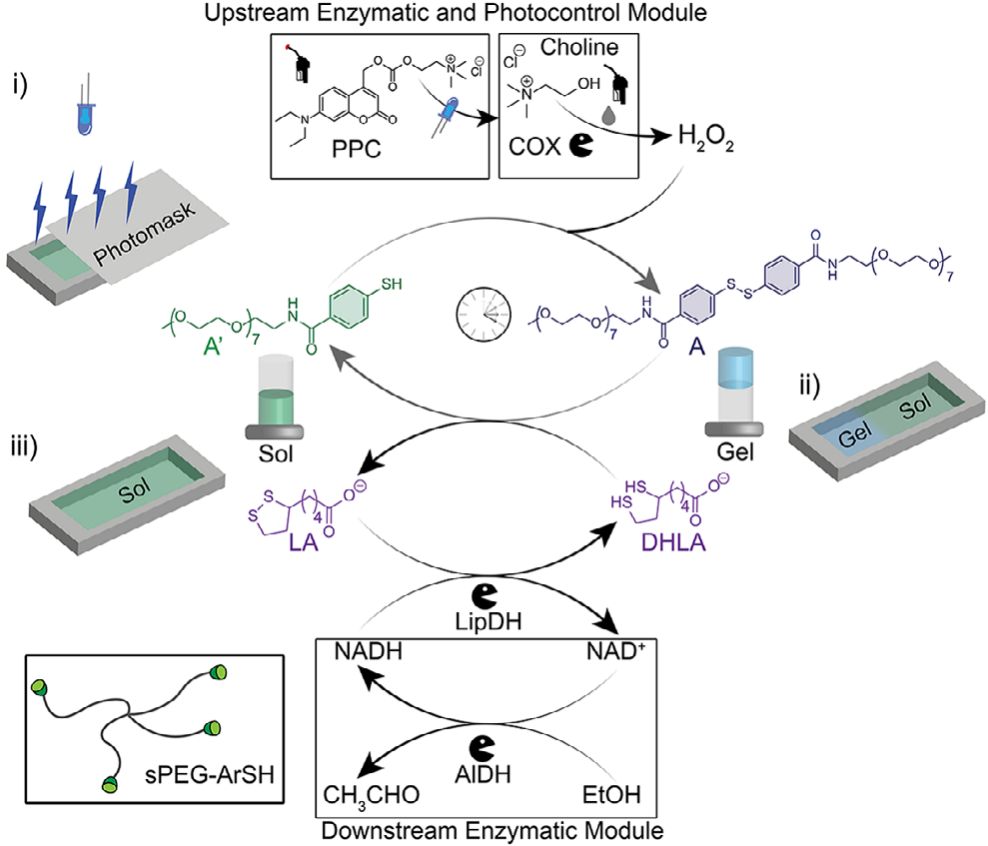
Fuel‐driven redox‐ERN with upstream and downstream enzymatic modules for photoinitiation and multiple refueling of thiol‐disulfide systems. Schematic shows spatial irradiation of sPEG‐ArSH solution to spatially activate the ERN (i) for dissipative hydrogel pattern formation (ii and iii).

Our approach for the pERN coupled to the dissipative formation of a thiol‐disulfide network with a quasi‐steady state (QSS) is illustrated in Figure 1. Upstream of the core ERN, we incorporate choline oxidase (COX) to enzymatically produce H_2_O_2_ from photoprotected choline^[^ ^68^ ^]^ which can be uncaged by blue light. We further demonstrate the downstream integration of alcohol dehydrogenase (AlDH) to replenish the oxidized NAD^+^ to NADH at the expense of ethanol. This downstream EM enhances ERN robustness, enabling multiple refueling cycles with lower [choline], challenging for reported redox‐based CRNs or ERNs.^[^ ^53^, ^54^, ^55^, ^56^, ^57^ ^]^ We utilize an aromatic thiol‐containing molecule (**A/A’**), terminated with polar groups for molecular solubility and aromatic units for tracking using HPLC, to standardize the ERN in solution. Finally, we will demonstrate how this photoinitiated, redox‐based network can be used to create spatiotemporal and transient hydrogels,^[^ ^28^, ^69^ ^]^ by photochemically fueling solutions containing aromatic thiol‐terminated star PEG polymers (sPEG‐ArSH). We provide insights into the processes at the sol/gel interface and its autonomous reversion via particle imaging velocimetry.

## Results and Discussion

We first emphasize our choice of an oxidase that is orthogonal to the reducing part of our ERN composed of lipoamide dehydrogenase (LipDH) and dihydrolipoic acid (DHLA). Widely studied oxidases, like glucose oxidase (GOX), were found to be inhibited in the presence of DHLA (Figure S1a). Alcohol oxidase (AOX) was found to inhibit the reducing half of the ERN (Figure S1b). To incorporate a functional oxidase upstream in the network, we finally selected COX, which recognizes choline primarily through electrostatic interactions^[^ ^70^ ^]^ and remains active in the presence of DHLA (Figure S1c).

**Figure 2:**
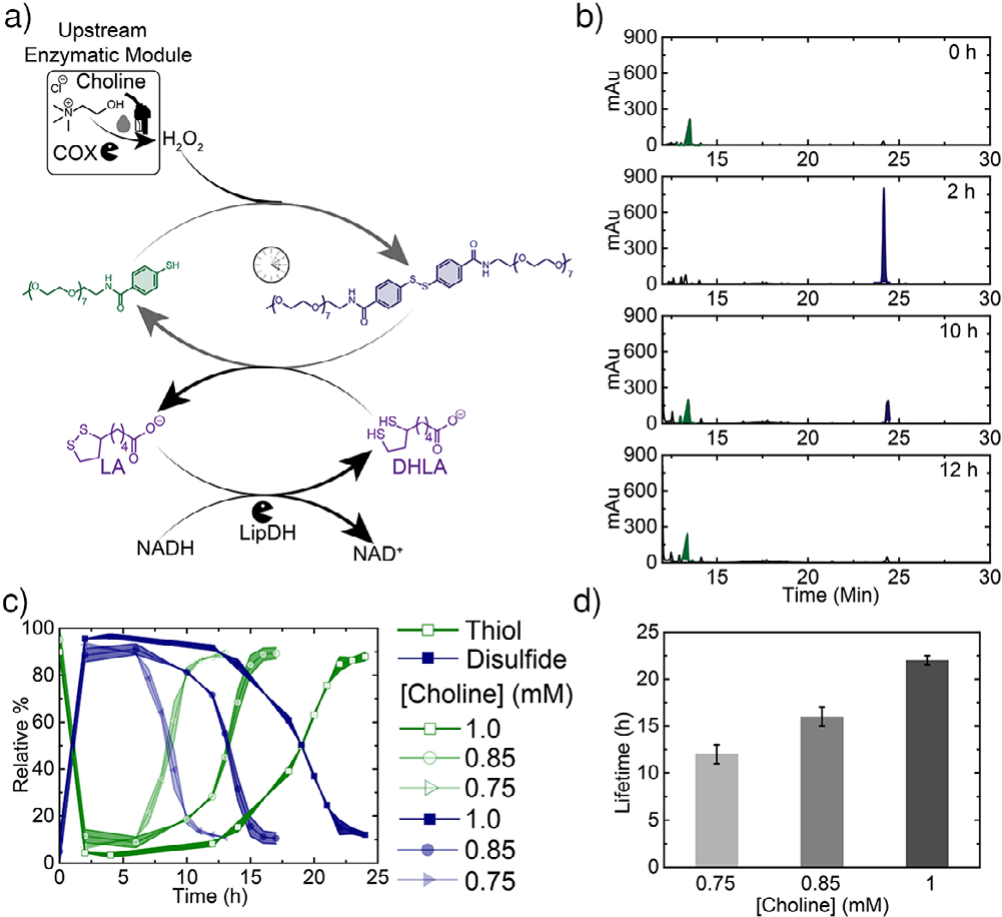
Choline fueled dissipative aromatic disulfide formation using ERN containing upstream EM. a) Scheme of redox ERN containing upstream EM. b) Time dependent HPLC chromatogram showing dissipative aromatic disulfide formation fueled by choline. c) and d) Programmable lifetime of aromatic disulfide at different [Choline]. The shaded area in c) and error bars in d) correspond to the standard deviation of duplicates. [**A**] = 0.1 mM, [NADH] = 6 mM, [DHLA] = 0.5 mM, [LipDH] = 5 mU µL^−1^, [COX] = 1.33 mU µL^−1^.

Using this setup, we assembled our first set of ERNs containing the upstream EM (Figure 2a). To establish the starting conditions, we reduced disulfide **A** to thiol **A'** with 0.5 mM DHLA. We then added 6 mM NADH, 5 mU µL^−1^ LipDH, and 1.33 mU µL^−1^ COX and initiated the ERN using 0.75 mM choline as fuel. Monitoring the ERN progress with HPLC (Figure 2b) reveals that the peak corresponding to **A’** (12 min) nearly vanishes within 2 h, with the appearance of a peak representing disulfide **A** (24 min). **A** remains in a QSS for 12 h before its peak intensity decreases, accompanied by a resurgence of the **A'** peak. This corresponds to fuel consumption and the takeover of the deactivation reaction. The lifetime of the QSS can be adjusted by altering [choline]. Increasing [choline] from 0.75 to 1 mM extends the lifetime from 12 to 32 h, demonstrating the temporal programmability of the system (Figure 2c,d; HPLC in Figure S2). We determined the lifetime of the system by the point at which the decrease in the **A** peak intensity became nearly constant.

**Figure 3:**
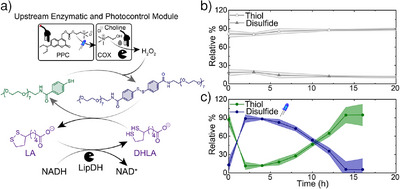
a) Schematic showing photochemically fueled dissipative aromatic disulfide formation. Relative % of thiol and disulfide of b) unirradiated solution, c) irradiated solution containing PPC. The shaded area in (b), c) corresponds to the standard deviation of duplicates. [**A**]=0.1 mM, [NADH] = 6 mM, [DHLA] = 0.5 mM, [LipDH] = 5 mU µL^−1^, [COX] = 1.33 mU µL^−1^, [PPC] = 0.5 mM.

To enable phototriggered behavior and ultimately spatial control, we introduce a 7‐(Diethylamino)‐4‐(hydroxymethyl) coumarin (DEACM)‐protected choline, that undergoes light‐induced uncaging at 420 nm, which is spectrally distinct from the NADH absorption. Our ERN containing this photoprotected choline (PPC) is completely dormant in the absence of light (Figure 3b; HPLC in Figure S3). To photo release the protected choline we irradiated it with 420 nm LED, where a 10 mW cm^−2^ LED intensity is sufficient to release 9 mM PPC (Figure S4). An essential synthetic step is the replacement of the iodide counterion to chloride, because iodide can be oxidized to iodine by H_2_O_2_, potentially disrupting the ERN by oxidizing NADH or the enzyme (HPLC in Figure S5a,b). With the PPC, we next attempted to photoinitiate the ERN (Figure 3a). To achieve this, **A** was first reduced to the thiol form **A'** using 0.5 mM DHLA. Subsequently, the system was supplemented with 5 mU µL^−1^ LipDH, 1.33 mU µL^−1^ COX, 6 mM NADH, and 0.5 mM PPC. We next irradiated the solution with a 420 nm LED at a power of 5 mW cm^−^^2^ for 30 min, aiming to photorelease 0.5 mM choline. Indeed, 2 h after the irradiation, the peak corresponding to disulfide **A** appears, along with a decrease in the peak intensity of thiol **A'** (Figure 3c; HPLC in Figure S5c). This state remains stable for 2 h in a QSS until the system exhausts its fuel, resulting in a decrease in the peak intensity of **A** and an increase in **A'**. The original ERN conditions regarding the balance of **A** to **A’** are restored after approximately 14 h, demonstrating that the system can be photoinitiated.

**Figure 4:**
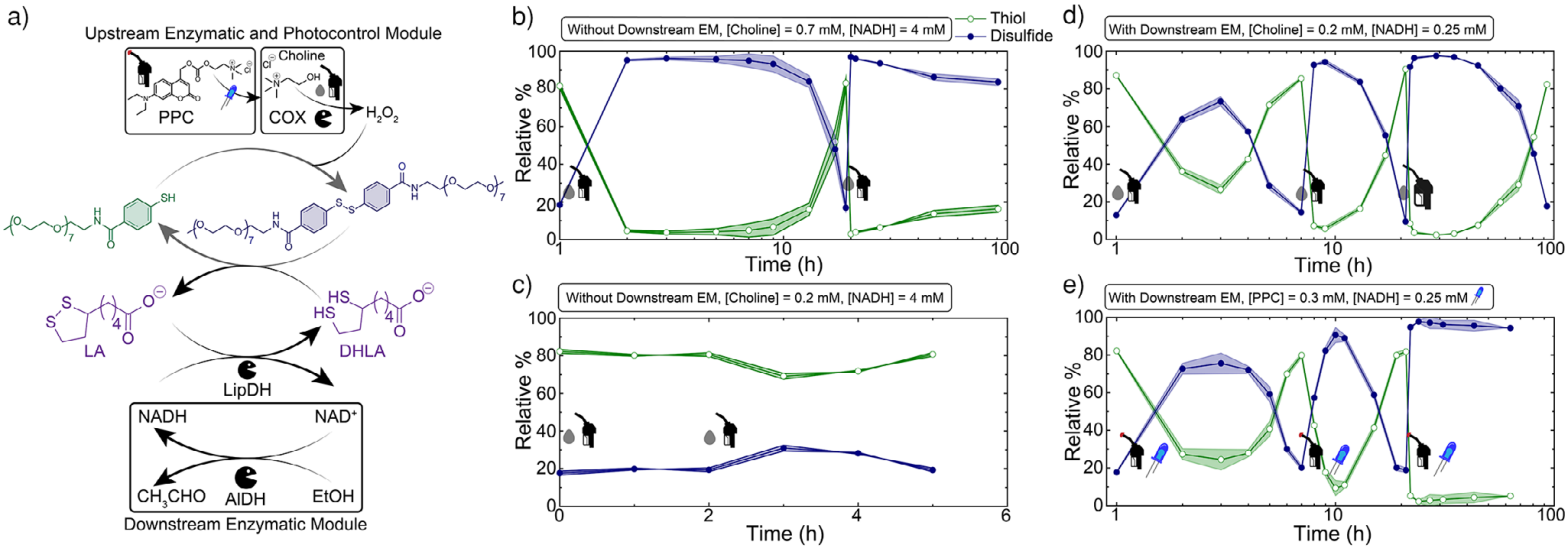
a) Schematic of redox ERN containing downstream EM for multiple refueling and formation of dissipative aromatic disulfide. Relative % of aromatic thiol and disulfide during multiple refueling when b) fueled with 0.7 mM, c) 0.2 mM choline without any downstream EM, and when fueled with d) 0.2 mM choline, e) 0.3 mM PPC in presence of downstream EM. The shaded area corresponds to standard deviation of duplicates. Conditions for (b), (c), are [**A**] = 0.1 mM, [NADH] = 4 mM, [DHLA] = 0.25 mM, [LipDH] = 5 mU µL^−1^, [COX] = 1.33 mU µL^−1^. Conditions for d and e are [**A**] = 0.1 mM, [NADH] = 0.25 mM, [DHLA] = 0.25 mM, [EtOH] = 100 mM, [LipDH] = 5 mU µL^−1^, [COX] = 1.33 mU µL^−1^, [AOX] = 6 mU µL^−1^.

One of the intriguing prospects for photofueling ERNs is the possibility of storage and on‐demand activation of photocaged fuels. This is however not possible for this particular photocaged choline as the carbonate linkage hydrolyzes with a half‐life of 10.5 h in our buffer conditions (Figure S6). One possible approach to prevent hydrolysis in aqueous medium is to protect choline with a nitrobenzene or coumarin‐based photoremovable group via a hydrolytically more stable ether linkage.^[^ ^71^, ^72^ ^]^ However, to still understand the robustness of the system, we first attempted refueling cycles with pure choline. Unfortunately, we observed that a second refueling cycle (0.7 mM choline) does not yield a transient response (Figure 4a,b). Although the produced H_2_O_2_ successfully oxidizes **A'** to **A**, the system does not revert back within 100 h, possibly due to the limited capacity of the enzyme to buffer the additional H_2_O_2_ generated. To address this, we refueled the system with a lower [choline] (0.2 mM; Figure 4c). However, this approach fails to produce any disulfide, likely because the rate of the deactivation exceeds that of the activation process.

To overcome this issue on a conceptual level, we installed a downstream module based on AlDH to regenerate NADH from NAD⁺ using ethanol (Figures 4a, S7). Further, we decreased [NADH] from 4 to 0.25 mM in the ERN which will slowly get replenished by AlDH. This provides additional kinetic control, helping in slowing down the deactivation reaction by slowly releasing NADH instead of the high concentration of NADH used in ERN conditions of Figure 4b,c. Following this rational, we reassembled the modified ERN using 0.25 mM DHLA, 0.25 mM NADH, 6 mU µL^−1^ of AlDH, and 100 mM ethanol, and then fueled the system with 0.2 mM choline. Time‐dependent HPLC chromatograms of the ERN demonstrate successful refueling for at least three cycles with 0.2 mM choline (Figure 4d). An increased lifetime occurs in subsequent cycles (Figure S8). Since there is no notable loss of enzymatic activity over time, both in presence and absence of DEACM (photo‐released chromophore) (Figure S9), we hypothesize the increased lifetime of subsequent cycles are likely due to the accumulation of waste products. To potentially observe more cycles, we increased the [LipDH] concentration from 5 to 8.3 mU µL^−1^. However, this adjustment failed to produce even the first transient cycle, due to an increased rate of the reducing half of the reaction cycle. Yet, this overall demonstrates a very beneficial repeated activation. Using this extended ERN, multiple photoactivation cycles are also possible by re‐addition of the photocaged choline (PPC) to deal with the hydrolysis issue (0.3 mM PPC; Figure 4e). Since higher PPC concentrations are needed to compensate for the slower photo‐generation of H₂O₂ as compared to directly adding choline to achieve transient states, only two activations are possible because of the totally increased H₂O₂ generation.

To explore spatial activation of the ERN within a material system, we next demonstrate spatiotemporally controlled hydrogelation using sPEG‐ArSH, which forms hydrogels through crosslinking driven by disulfide bond formation (Figure S10, rheology). Initially, we examined dissipative hydrogel formation using choline as fuel. For this system, we assembled 2.5 mM of sPEG‐ArSH, along with 60 mM of NADH, 2.5 mM DHLA, 0.03 U µL^−1^ of COX, and varying concentrations of LipDH, adjusted to account for the increased aromatic thiol end‐group content compared to the solution studies, and fueled the system by adding 9 mM choline. The vial inversion test (Figure S11, Video S1) shows a low‐viscosity solution prior to choline addition, which transforms into a gel due to enzymatic H_2_O_2_ production. Due to unavoidable issues of limited oxygen diffusion into the system during in situ rheological measurement (stoichiometric O_2_ is needed by COX, sealing is needed to prevent drying of the hydrogel), we evaluated the sol‐gel‐sol transition by observing the videos at the point where the gel flows to the bottom of the vial. The systems show only a mild dependence of the lifetime on [LipDH]. Ex situ rheology further confirms the sol (initial state) to gel (QSS) to sol (final state) transition driven by consumption of choline (rheology in Figure S11c‐e).

**Figure 5:**
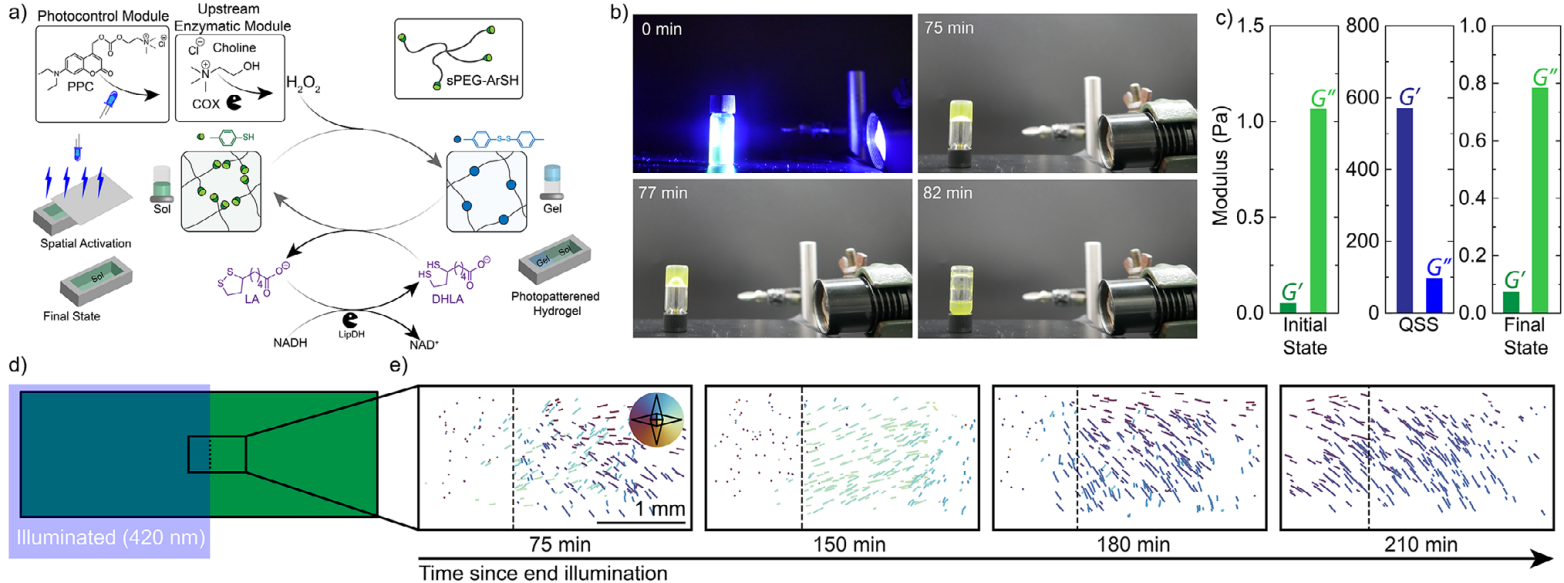
a) Schematic showing integration and photoactivation of redox ERN containing upstream enzymatic module to sPEG‐ArSH for spatiotemporally controlled dissipative hydrogel formation. b) Snapshot series of the photoactivated dissipative hydrogels demonstrates melting of the formed hydrogel upon photoactivation after 82 min. c) Respective modulus in the sol state (no light), QSS (*t* =  75 min), and at the end of the cycle (*t* = 12 h) at a frequency of 1 Hz and strain = 1%. d) Sketch of the sample chamber indicating where microscopy takes place. e) Particle traces over a 40 s interval at 0.5 fps at different times after illumination. Each line indicates a particle trace, color‐coded for the direction of travel (see inset for directions). The dotted line indicates the approximate location of the edge of illumination. ([sPEG‐ArSH] = 2.5 mM, [DHLA] = 2.5 mM, [NADH] = 60 mM, [LipDH] = 0.025 U µL^−1^, [COX] = 0.03 U µL^−1^, [PPC] = 9 mM).

Next, to achieve photoactivated dissipative hydrogels with sPEG‐ArSH, we replaced direct addition of choline by PPC (9 mM), followed by irradiation with 10 mW cm^−^^2^ 420 nm light for 30 min to release the choline (photouncaging studies in Figure S4). Time‐dependent snapshots confirm the hydrogel formation after 75 min, as observed in the vial inversion test (Figure 5a,b, Video S2). The gel remains in a QSS until about 80 min before flowing to the bottom of the vial. Ex situ rheology further confirms hydrogel formation in the QSS, showing a higher loss modulus (*G’*) than storage modulus (*G’’*) (rheology in Figure S12). Notably, the *G'* obtained by photoreleasing choline is lower than that achieved through direct choline fueling. This is due to the slow release of choline during photodeprotection compared to direct addition of choline as a fuel.

To showcase the spatial control in photoactivated dissipative hydrogels, we filled a custom PDMS‐based reactor with an sPEG‐ArSH solution containing 2.5 mM DHLA, 60 mM NADH, 0.025 U µL^−1^ LipDH, 0.03 U µL^−1^ COX, and 9 mM PPC that can be irradiated only on one part. Additionally, we dispersed fluorescent particles (1–5 µm in diameter) into the solution to enable particle tracking to probe the spatiotemporal formation of the hydrogel. Using this setup, we irradiated the solution, covering half of it with a photomask (Figure 5a inset), using a 420 nm LED at an intensity of 10 mW cm^−^^2^ for 30 min. Subsequently, we started tracking the movement of the dispersed particles after 75 min at the interface between the irradiated and non‐irradiated regions (Figure 5d,e, Video S3) in 15 min intervals. The particle traces show that the particles are highly dynamic in the non‐irradiated area, whereas particles are static in the irradiated area, confined by the surrounding hydrogel network. This confirms the spatial activation of the ERN and hydrogel formation. Such hydrogels can even be developed by washing away the non‐gelled part (Figure S13).

Interestingly, particle tracking reveals that the spatially activated gel dissolves from the interface between gelled and non‐gelled regions, with the process gradually progressing inward (to the left). After approx. 3 h, all particles are dynamic, corresponding to a full gel‐to‐sol transition, and underscoring the spatiotemporal nature of hydrogel formation. On a microscopic level, we note that particles move predominantly toward the hydrogelled region close to the gelled and non‐gelled domains during melting. We speculate this occurs due to a gradient of DHLA, which is higher in the non‐irradiated region compared to the irradiated area. This diffusophoretic movement of DHLA may drive the net migration of particles toward the hydrogelled portion.

## Conclusion

In summary, we presented a versatile chemically fueled cascaded redox‐ERN incorporating new and important upstream and downstream EMs, enabling robust photoactivation and refueling to drive dissipative aromatic disulfide formation. We demonstrated how COX can be integrated upstream in the ERN to activate the system using choline as a fuel, enabling a [choline]‐dependent lifetime. To achieve photoinitiation of the ERN, we employed a photoprotected choline using a coumarin moiety, which allows choline to be photoreleased and the ERN to be activated by blue light. Additionally, we incorporated a downstream AlDH to regenerate NADH from NAD^+^ using ethanol, thereby slowing down the reducing half of the reaction cycle and enabling system refueling with lower fuel concentrations. In contrast to our earlier redox‐CRN,^[^ ^67^ ^]^ this hierarchical ERN system now has two additional modules upstream and downstream to enable robust photoactivation and facilitate system refueling.

To demonstrate the utility of this photoactivated ERN, we coupled it with sPEG‐ArSH to achieve photoinitiated dissipative hydrogel formation. At the material level, we demonstrated the spatial activation of the ERN, enabling spatiotemporally controlled hydrogel formation. Particle tracking provided deep insights in the dissolution process and flow occurring in the system. This redox‐based ERN provides a foundation for spatiotemporal and dissipative activation of thiol‐disulfide‐based materials and systems. In future, the upstream and downstream EMs can be photocontrolled using different wavelengths of light, allowing for complex spatiotemporal responses or spatial regulation (upregulation or downregulation) of system behavior when integrated into material systems.

## Supporting Information

The authors have cited additional references within the Supporting Information.^[^ ^73^ ^]^

## Conflict of Interests

The authors declare no conflict of interest.

## Supporting information



Supplementary Information

Supplementary Information

Supplementary Information

Supplementary Information

Supplementary Information

## Data Availability

The data that support the findings of this study are available from the corresponding author upon reasonable request.
