# Supplementary material for: Photoactivated Enzymatic Reaction Network Enables Spatiotemporal Programming of Thiol/Disulfide Redox Systems
Source: Angew Chem Int Ed Engl. 2025 May 5;64(25):e202503822. doi: 10.1002/anie.202503822 (PMC12171324; doi:10.1002/anie.202503822)
Supplement: Supplementary file 4 — Supplementary Information [file ANIE-64-e202503822-s005.pdf]

Supporting Information for:

Photoactivated Enzymatic Reaction Network Enables Spatiotemporal Programming of Thiol/Disulfide Redox Systems

Aritra Sarkar, Piet J. M. Swinkels, Lea Duttenhofer, Pol Besenius, Andreas Walther*

Department of Chemistry, University of Mainz, Duesbergweg 10-14, 55128 Mainz, Germany-560064.

Contents

A) Experimental sections 2

A.1. General methods 2

A.2. Experimental procedures 2

A.3. Synthetic schemes and procedures 4

A.4. Supporting figures 7

B) Characterization spectra 17

C) References 19

A) Experimental sections

A.1. General methods

Materials: 1-Hydroxybenzotriazole hydrate, N, N-diisopropylethylamine, aniline, DL-α-dihydro lipoic acid, (R)-6,8-dimercaptooctanoic acid, β-nicotinamide adenine dinucleotide reduced disodium salt hydrate was purchased from Sigma Aldrich. 4-arm Poly(ethylene glycol) amine 20 KD was purchased from SINOPEG. HPLC grade acetonitrile was purchased from VWR Germany. Choline chloride (98%), Dowex® 22 Cl Form, choline oxidase from arthrobacter sp., bis(trichloromethyl) carbonate, methyl Iodide, *tert*-Butyl mercaptan were purchased from Sigma Aldrich. 7-(Diethylamino)-4-(hydroxymethyl)coumarin (DEACM) was purchased from TCI. Native porcine lipoamide dehydrogenase (LipDH) (25 U/mg protein) was purchased from Creative Enzymes, USA. All chemicals and enzymes were used as received.

NMR measurements: NMR spectra were recorded with a Bruker AVANCE 400 (400 MHz) NMR spectrometer. Splitting patterns are designated as s, singlet; d, doublet; bs, broad singlet; m, multiplet; t, triplet.

Mass spectrometry: ESI measurements were executed on an agilent 6545QTOF-MS device.

High-Performance liquid chromatography: A Thermo Fischer Scientific Dionex Ultimate 3000 HPLC system was used and chromatograms were processed using the chromeleon data system. Small molecule samples were dissolved in 0.1 M (pH = 7.4) phosphate buffer (PB) and separated by using a gradient from 100% water to 100% acetonitrile. A Nucleosil 100-5-C18, 4.6 × 250 mm reversed-phase column was used. The samples (thiol and the disulfide) were detected at 254 nm wavelength using a diode array detector. The relative percentage of thiol and disulfide was calculated using in build program in chromeleon chromatography studio.

Rheology: An Anton Paar Modular Compact Rheometer MCR 302 equipped with a 25 mm plate-plate geometry was used for rheological measurements. Analysis of the data was done with the rheocompass software.

Irradiation setup: A mightex LED of wavelength 420 nm with an output of 270 mW was used. The power was adjusted using the BioLED light source control module from mightex. The irradiation light intensity was measured using a power meter in mW/cm^2.

Microscopy and particle tracking: To observe the spatiotemporal gelling of the sample (Fig. 5 in the main text), we mixed commercial tracer particles (cospheric, 1-5 µm in diameter, 1.3 g/mL, magenta) with the samples at approximately 0.16 vol%. These particles are chosen such that their excitation/emission spectra do not overlap with any other components in the system, especially the photocontrol module on the blue end of the visible light spectrum. Observation took place in a home-built automated microscopy setup to cover a large area of the sample, with a 520-35 excitation filter and a 620-52 emission filter. We recorded 20 images at 0.5 fps every 15 minutes to probe particle movement over time. Tracking was done using the *trackpy* particle tracking module for Python[S1]. Raw movies of particle movement are available as Supplementary Video File 3.

A.2. Experimental procedures

Protocol I: Sample preparation protocol for transient oxidation of A' using upstream enzymatic module: To investigate the transient oxidation of **A'** using the upstream enzymatic module, a solution containing 0.1 mM **A**, 0.5 mM (±)-DHLA, 6 mM NADH, 5 mU/µL LipDH, and 1.3 mU/µL choline oxidase was prepared by injecting appropriate volume into PB buffer containing 1 wt% BSA from a stock solution of 5×10^{-3} M **A**, 0.1 M NADH, 1 U/µL of choline oxidase (COX), and 0.5 U/µL LipDH, prepared in 0.1 M phosphate buffer (PB) (pH = 7.4) containing

1 wt% of BSA and 0.5 M (±)-DHLA dissolved in dimethyl sulfoxide (DMSO). To this solution, 0.75 mM, 0.8 mM, and 1 mM choline chloride were added from a stock solution of 0.5 M choline chloride in 0.1 M PB (pH = 7.4) containing 1 wt% of BSA to vary the fuel concentration. The final volume of the solution was 1.5 mL.

Protocol II: Sample preparation protocol for transient oxidation of A' using upstream photocontrol module: For transient oxidation of **A'**, a solution containing 0.1 mM reduced **A**, 6 mM NADH, 0.5 mM (±)-DHLA, 5 mU/μL LipDH, 1.33 mU/μL COX, and 0.5 mM photoprotected choline (PPC, synthetic scheme S4) was prepared by injecting the stock solution of **A** ([**A**] = 0.5 mM), NADH ([NADH] = 0.1 M), LipDH ([LipDH] = 0.5 U/μL), COX (1 mU/μL), and PPC (0.1 M) dissolved in 0.1 M PB containing 1 mg/mL BSA, and 0.5 M (±)-DHLA dissolved in DMSO. The solution was irradiated for 30 minutes with 420 nm LED with a power of 5 mW/cm^2 (measured using a power meter) to initiate the ERN. The final volume of the solution was 1.5 mL.

Protocol III: Sample preparation protocol for transient oxidation of A' and multiple refueling using choline as fuel with upstream and downstream enzymatic module: For transient oxidation of **A'**, a solution containing 0.1 mM reduced **A**, 0.25 mM NADH, 0.25 mM (R)-DHLA, 5 mU/μL LipDH, 1.3 mU/μL COX, 6 mU/μl alcohol dehydrogenase (AlDH), and 100 mM ethanol in 0.1 M PB containing 1 mg/mL BSA was prepared by injecting the stock solution of **A** ([**A**] = 0.5 mM), NADH ([NADH] = 0.1 M), LipDH ([LipDH] = 0.5 U/μL), COX (1 U/μL), alcohol dehydrogenase (AlDH) (1 U/μL) dissolved in 0.1 M PB containing 1 mg/mL BSA and 0.5 M (R)-DHLA dissolved in DMSO. The solution was fueled multiple times using 0.2 mM of choline from a stock solution of 0.1 M choline in 0.1 M PB containing 1 mg/mL BSA. The final volume of the solution was 1.5 mL. For multiple refueling experiment we used R-DHLA instead of racemic mixture of DHLA. Considering during the second batch of fuel addition we lose half of the racemic mixture of DHLA during enzymatic conversion we decided to use R-DHLA for this experiment.

Protocol IV: Sample preparation protocol for transient oxidation of A' and multiple refueling using PPC as fuel with upstream photocontrol module and downstream enzymatic module: For transient oxidation of **A'**, a solution containing 0.1 mM reduced **A**, 0.25 mM NADH, 0.25 mM (R)-DHLA, 5 mU/μL LipDH, 1.3 mU/μL COX, 6 mU/μl AlDH, and 100 mM ethanol in 0.1 M PB containing 1 mg/mL BSA was prepared by injecting the stock solution of **A** ([**A**] = 0.5 mM), NADH ([NADH] = 0.1 M), LipDH ([LipDH] = 0.5 U/μL), COX (1 U/μL), AlDH (1 U/μL) in 0.1 M PB containing 1 mg/mL BSA and 0.5 M (R)-DHLA dissolved in DMSO. The solution was fueled multiple times using 0.3 mM of PPC from a stock solution of 0.25 M PPC in 0.1 M PB containing 1 mg/mL BSA. After each addition of PPC the solution was irradiated for 30 minutes using 420 nm LED with a power of 5 mW/cm^2 to initiate the ERN. The final volume of the solution was 1.5 mL. For multiple refueling experiment we used R-DHLA instead of racemic mixture of DHLA. Considering during the second batch of fuel addition we lose half of the racemic mixture of DHLA during enzymatic conversion we decided to use R-DHLA for this experiment.

Protocol V: Sample preparation protocol for transient hydrogelation of sPEG-ArSH using choline oxidase and choline as a fuel: To obtain a transient hydrogel, 15 mg of sPEG-ArSH (synthetic scheme S6, and S7), was dissolved in 0.1 M PB containing 1 mg/mL BSA, and in it, 60 mM NADH, varying concentration of LipDH, 0.03 U/μL COX, 2.5 mM (±)-DHLA was added from a stock solution of 1 M NADH, 0.5 mU/μL LipDH, 1 mU/μL COX dissolved in 0.1 M PB containing 1 mg/mL BSA and 1 M (±)-DHLA dissolved in DMSO. In it 9 mM choline was

added from a stock solution of 0.5 M choline dissolved in 0.1 M PB containing 1 mg/mL BSA to initiate the transient hydrogel formation. The final volume of the solution was 300 μL.

Protocol VI: Sample preparation protocol for transient hydrogelation of sPEG-ArSH using choline oxidase and photoprotected choline as a fuel: To obtain a transient hydrogel which can be initiated using a light trigger, 15 mg of sPEG-ArSH, was dissolved in 0.1 M PB containing 1 mg/mL BSA, and in it, 60 mM NADH, 0.025 U/μL of LipDH, 0.03 U/μL COX, 2.5 mM (±)-DHLA, 9 mM of PPC was added from a stock solution of 1 M NADH, 0.5 mU/μL LipDH, 1 mU/μL COX dissolved in 0.1 M PB containing 1 mg/mL BSA and 1 M (±)-DHLA dissolved in DMSO. In it 9 mM PPC was added from a stock solution of 0.25 M PPC dissolved in 0.1 M PB containing 1 mg/mL BSA. The solution was irradiated with a 420 nm LED with an intensity of 10, mW/cm^2 to initiate the transient hydrogel formation. The final volume of the solution was 300 μL.

A.3. Synthetic schemes and procedures

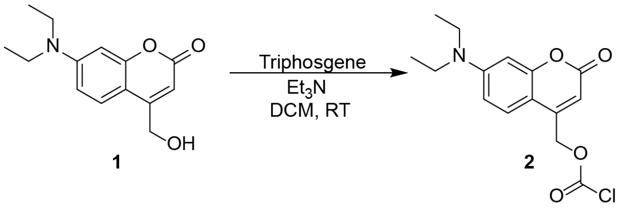

Scheme S1. Synthetic route to molecule **2.**

Synthesis of **2**: 0.081 g of bis(trichloromethyl)carbonate (0.27 mmol) was dissolved in dry CH_2Cl_2 (5 mL) and cooled in an ice bath. 200 mg of 7-(diethylamino)-4-(hydroxymethyl)coumarin (0.8 mmol) was slowly added while stirring, and then a mixed solution of triethylamine (0.08 g, 0.8 mmol) and CH_2Cl_2 (5 mL) was added dropwise at 0-5 °C. After the addition, the reaction mixture was stirred for 4 h at room temperature. The crude mixture was washed with water, and then the CH_2Cl_2 layer was dried with anhydrous Na_2SO_4, filtered, and concentrated to give compound **2**. The reaction mixture was used as such in the next step without further treatment.

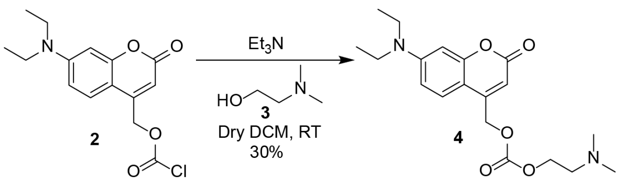

Scheme S2. Synthetic route to molecule **4.**

Synthesis of **4**: To a 0.1 g solution of **3** (0.96 mmol) and 0.147 g triethylamine (1.45 mmol) in dry CH_2Cl_2 (5 mL) was added dropwise a solution of 300 mg of **2** (0.09 mmol) in CH_2Cl_2 at 0 °C. After stirring at room temperature for 12 h the reaction mixture was diluted with CH_2Cl_2 and washed with HCl (1 N), saturated $NaHCO_3$, and finally with saturated NaCl solution. The collected organic layer was dried with Na_2SO_4 and the solvent was removed under vacuum. The crude product was purified by column chromatography using a solvent gradient ranging from pure chloroform to 10% methanol in chloroform to get 60 mg of the pure product. Yield = 17%; ^1H NMR: (400 MHz, $CDCl_3$, ppm): δ = 7.32 (d, 1H, H^{Ar}), 6.63 (dd, 1H, H^{Ar}), 6.52 (d, 1H, H^{Ar}), 6.13 (s, 1H, CHCO), 5.31 (d, 2H, OCH_2),

4.77 (m, 2H, OCH2), 3.43 (t, 6H, NCH_2), 2.92 (d, 6H, $N(CH_3)_2$), 1.22 (t, 6H, NCH_2CH_3); ^{13}C NMR: (100 MHz, $CDCl_3$, ppm): 161.60, 156.23, 153.76, 148.25, 124.53, 65.37, 62.56, 56.06, 45.15, 43.70, 12.33; HRMS (ESI) m/z: calculated for $C_{19}H_{26}N_2O_5$: 362.1842; found: 385.1732 $[M+Na]^+$.

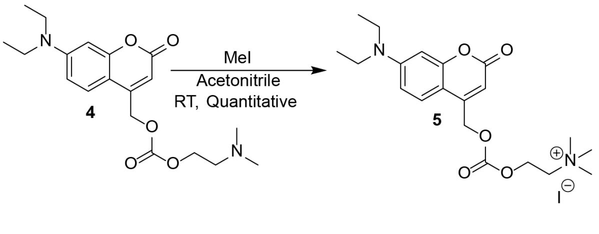

Scheme S3. Synthetic route to molecule **5.**

Synthesis of **5**: 60 mg of **4** (0.0165 mmol) was suspended in 10 mL of acetonitrile and 0.234 g of MeI (0.165 mmol) was added and stirred for 1 h. After 1 h, the completion of the reaction was ensured using TLC. After completion, the excess acetonitrile and MeI were evaporated under reduced pressure, and the crude mixture was dissolved in water and freeze-dried to get 82 mg of **5** as a yellow solid. Yield: Quantitative.

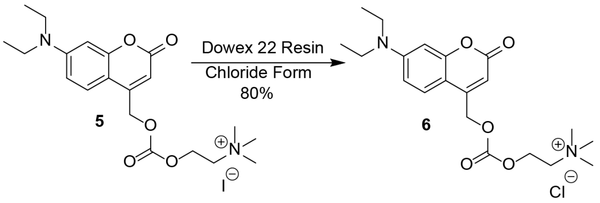

Scheme S4. Synthetic route to molecule **6 (PPC).**

Synthesis of **6**: To exchange iodide with chloride ion, Dowex® 22 resin (chloride form) was first washed with MilliQ water in a flask, to remove any fine particles. The resin (1 g of resin for 0.1 g of compound) was loaded into the column and activated with HCl 1N. After further washes, first with water and then with methanol, compound **5** was dissolved in methanol and then loaded onto the column, using pure methanol as eluent. The pure product **6** was collected with 80% yield. ^1H NMR: (400 MHz, DMSO-d_6, ppm): δ = 7.48 (d, 1H, H^{Ar}), 6.71 (dd, 1H, H^{Ar}), 6.56 (d, 1H, H^{Ar}), 6.07 (s, 1H, CHCO), 5.39 (d, 2H, OCH_2), 4.61 (m, 2H), 3.74 (m, 2H, $CH2^{OEG}$), 3.44 (q, 4H, NCH_2), 3.14 (s, 9H, $N(CH_3)_3$), 1.12 (t, 6H, CH_2CH_3); ^{13}C NMR: (100 MHz, DMSO-d_6, ppm): 160.99, 156.27, 153.82, 150.15, 126.08, 105.91, 65.35, 64.00, 62.38, 53.31, 44.60, 12.72. HRMS (ESI) m/z: calculated for $C_{20}H_{29}ClN_2O_5$: 412.1765; found: 377.2070 $[M+H]^+$.

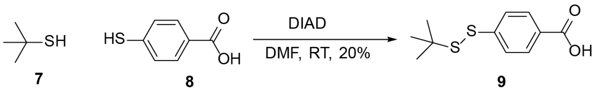

Scheme S5. Synthetic route to molecule **9**.

Synthesis of **9**: 1.17 g of **7** (12.97 mmol) and 1 g of **8** (6.49 mmol) was dissolved in 10 mL DMF and 2.62 g of diisopropyl azodicarboxylate (DIAD) was added. The reaction mixture was stirred overnight. After this, the solution was evaporated under reduced pressure and the crude mixture was purified using column chromatography with a gradient ranging from pure chloroform to 5% methanol in chloroform to get 320 mg of the pure product. Yield: 20%. ^1H NMR: (400 MHz, DMSO-d_6, ppm): δ = 7.91 (d, 2H, H^{Ar}), 7.68 (d, 2H, H^{Ar}), 1.29 (s,

9H, $(CH3)_3C$); ^{13}C NMR: (100 MHz, DMSO-d_6, ppm): 167.24, 144.02, 130.36, 129.19, 125.92, 68.28, 50.17, 29.81, 22.37. HRMS (ESI) m/z: calculated for $C_{11}H_{14}S_2O_2$: 242.0435; found: 241.0370 $[M\text{-}H]^-$.

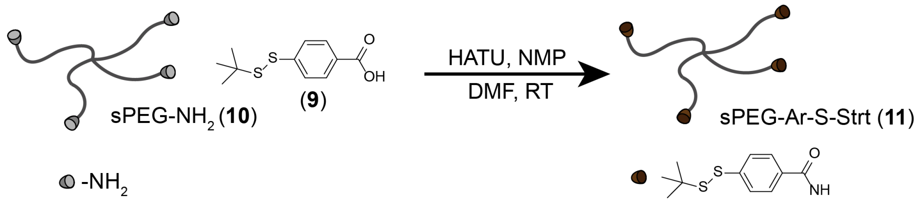

Scheme S6. Synthetic route to **sPEG-Ar-S-Strt.**

Synthesis of **sPEG-Ar-S-Strt (11)**: **9** (0.096 g, 0.4 mmol) and HATU (0.152 mg, 0.4 mmol) were dissolved in 6 mL anhydrous DMF. To this solution, N-Methylmorpholine (0.202 g, 2.00 mmol) was added and reacted for 30 min at room temperature. In parallel, 4-Arm-PEG-NH_2HCl (20 $kgmol^{-1}$) (1.00 g, 0.050 mmol) was dissolved in 3 mL anhydrous DMF and was added to the activated DBCO-COOH/HATU. The resulting mixture was stirred overnight at room temperature. The reaction was concentrated under reduced pressure, dissolved in MilliQ-water, dialyzed against Milli-Q water for 48 h (MWCO ≈ 6–8 kDa), filtered, and lyophilized. The product was obtained as a beige powder (0.95 g, 95%, degree of functionalization >95%)

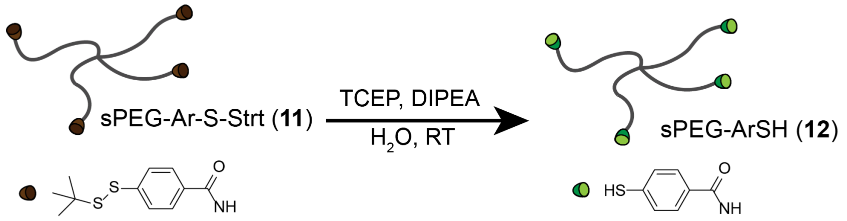

Scheme S7. Synthetic route to **sPEG-ArSH.**

Synthesis of **sPEG-ArSH (12)**: **11** (0.76 g, 0.038 mmol) was dissolved in water, followed by the addition of 1.08 g of Tris(2-carboxyethyl)phosphine hydrochloride (TCEP, 3.8 mmol) and 0.49 g of DIPEA (3.8 mmol). The reaction mixture was stirred for 1 day after which the solution was dialyzed against water for 48 h (MWCO ≈ 6–8 kDa), filtered, and lyophilized. The product was obtained as a beige powder (0.71 g, 94%, degree of functionalization >95%).

A.4. Supporting figures

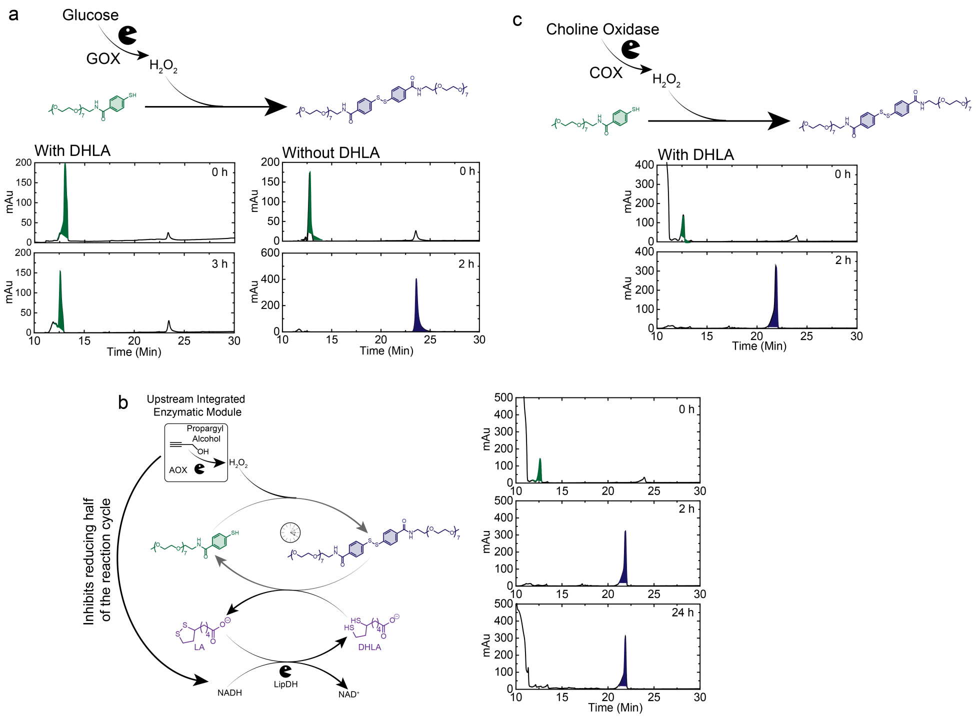

Figure S1. Compatibility of two different enzymes in the reaction cycle and the choice of oxidase. (a) Time dependent HPLC chromatograms demonstrating inhibition of glucose oxidase (GOX) activity in presence of DHLA ([**A**] = 0.1 mM, [DHLA] = 0.5 mM, glucose = 1 mM, GOX = 1.33 mU/μL). (b) Schematic representation and time dependent HPLC chromatograms demonstrating oxidation of **A'** to **A** using alcohol oxidase (AOX) and propargyl alcohol in presence of LipDH and DHLA, however the upstream enzymatic module inhibits reducing half of the reaction network due to which **A** does not reduce back with time ([**A**] = 0.1 mM, [DHLA] = 0.5 mM, [NADH] = 6 mM, [LiPDH] = 5 mU/μL, [AOX] = 1.33 mU/μL, [propargyl alcohol] = 0.75 mM). (c) Time-dependent HPLC chromatograms demonstrating successful oxidation of **A'** to **A** using choline oxidase (COX) and choline in presence of DHLA ([**A**] = 0.1 mM, [DHLA] = 0.5 mM, [choline] = 0.75 mM, [COX] = 1.33 mU/μL).

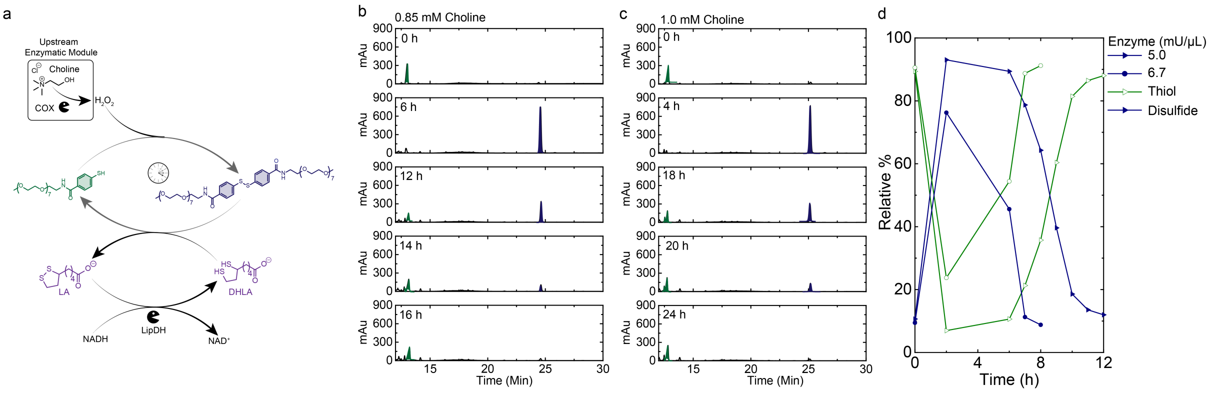

Figure S2. (a) Schematic depiction of dissipative aromatic disulfide formation using redox-ERN (enzymatic reaction network) with upstream integrated enzymatic module (COX and choline)**.** Time-dependent HPLC chromatogram demonstrating the dissipative aromatic disulfide formation on the addition of (b) 0.85 mM of choline and (c) 1.0 mM of choline to the **A'** solution containing dormant orthogonal enzymatic reducing environment and upstream COX. ([**A'**] = 0.1 mM, [DHLA] = 0.5 mM, [NADH] = 6 mM, [LipDH] = 5 mU/µL, [COX] = 1.33 mU/µL). (d) Programmable lifetime of the system using varying enzyme concentration ([**A'**] = 0.1 mM, [DHLA] = 0.5 mM, [NADH] = 6 mM, [COX] = 1.33 mU/µL, [Choline] = 0.75 mM).

Note: Time-dependent HPLC chromatogram depicts the fuel-dependent lifetime of the dissipative disulfides, where a higher concentration of choline produces higher amount of H_2O_2 which leads to an increased lifetime of the disulfides. The lifetime of the system can also be programmed by changing the [LiPDH] concentration where an increase in [LipDH] leads to faster deactivation reaction and thus lower lifetime of the disulfides.

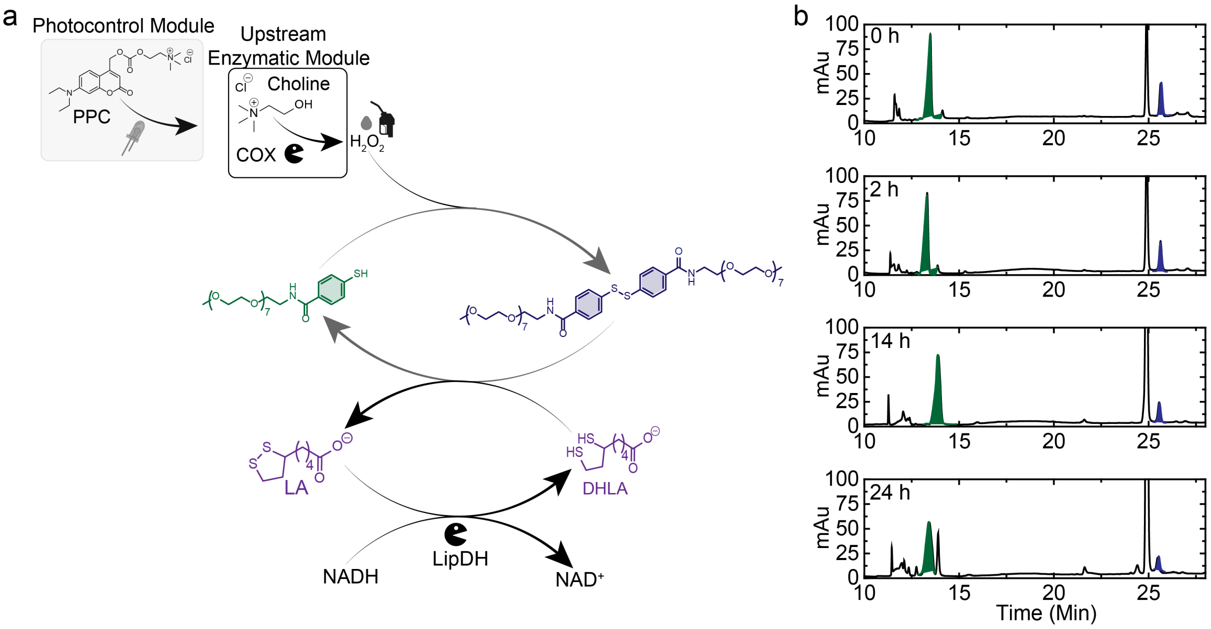

Figure S3. Time-dependent HPLC chromatogram showing the absence of **A** over 24 h without light irradiation, confirming dormancy of the system without photostimulation. ([**A**] = 0.1 mM, [NADH] = 6 mM, [DHLA] = 0.5 mM, [COX] = 1.33 mU/µL, [LipDH] = 5 mU/µL).

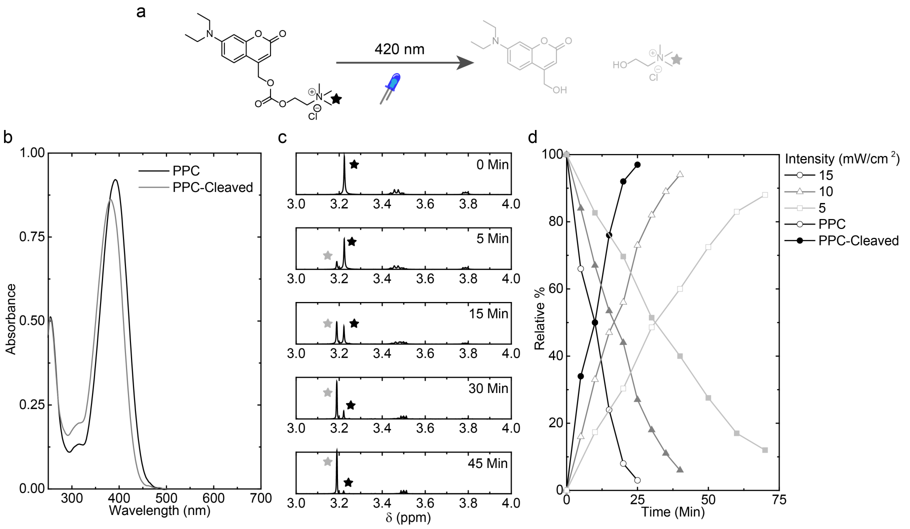

Figure S4. (a) Schematic showing photo deprotection of PPC using blue light irradiation (420 nm). (b) Absorption spectra of PPC before and after 30 minutes irradiation with 5 mW/cm^2 blue LED ([PPC] = 0.05 mM, pH = 7.4 PB containing 1 wt% BSA). (c) Time dependent ^{1}H-NMR spectra of 9 mM PPC with 10 mW/cm^2 intensity blue light irradiation demonstrate shifts in δ (ppm) of -N(Me)$_3$ peak upon photocleavage. The NMR spectra show 9 mM PPC could be successfully photocleaved using 30 minutes irradiation of 10 mW blue LED. (d) Relative percentage of PPC and photoreleased PPC (PPC-Cleaved) obtained from time dependent ^{1}H-NMR when irradiated with different intensity of 420 nm LED. (D_2O/Acetonitrile-d$_3$, 9/1 (*v*/*v*)).

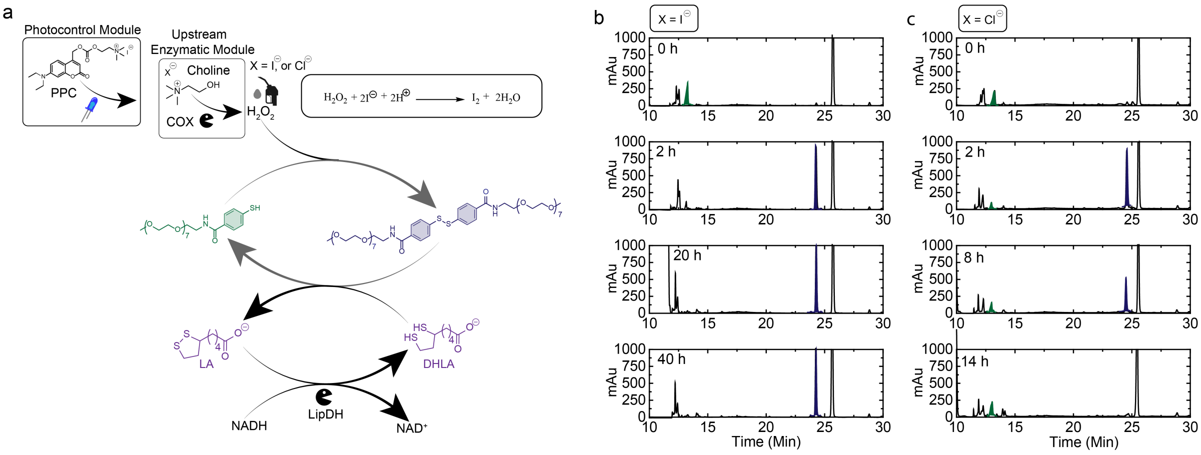

Figure S5. (a) Schematic showing photoactivation of the ERN for dissipative aromatic disulfide formation using PPC with iodide or chloride as a counter ion. The inset of the schematic shows oxidation of iodide to iodine with enzymatically produced H_2O_2. Time dependent HPLC chromatogram of photoactivated ERN using PPC with (b) iodide as a counter ion demonstrates oxidation of **A'** to **A**, which does not reduce back over time, indicating failure of the reducing half of the reaction cycle, and (c) chloride as a counterion which shows successful formation of dissipative disulfide (**A**) which reduces back over time. The HPLC peaks of **A**' and **A** is marked with green and blue color, respectively. The peak above 25 min represents the photoreleased hydroxy coumarin moiety. ([**A**] = 0.1 mM, [DHLA] = 0.5 mM, [NADH] = 6 mM, [COX] = 1.33 mU/μL, LipDH = 5 mU/μL, [PPC-I] = [PPC-Cl] = 0.5 mM).

Note: The PPC with iodide as a counter ion inhibits the reaction network. We suggest the enzymatically produced H_2O_2 reacts with iodide to produce iodine after photo-deprotection, which is a strong oxidant and might be oxidizing the enzyme or the NADH inhibiting the reducing half of the reaction cycle.

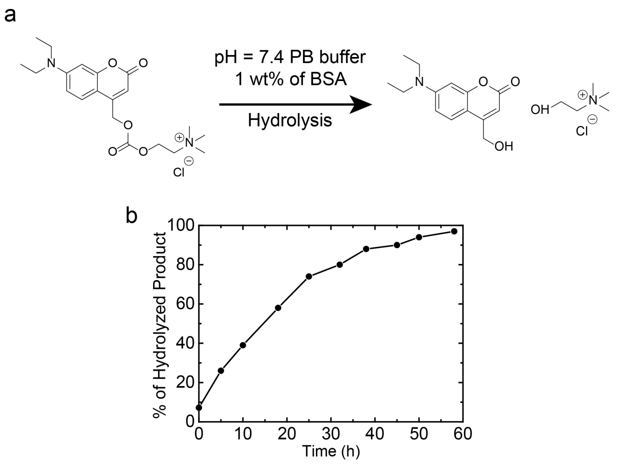

Figure S6. (a) Schematic depicting hydrolysis of PPC in PB buffer solution. (b) Relative % of hydrolyzed product in pH = 7.4 PB buffer containing 1 wt% of BSA monitored using HPLC shows a a half-life of 10.5 h.

Note: The carbonate group of PPC undergoes spontaneous hydrolysis in PB buffer, catalyzed by phosphate groups. To accurately determine the half-life of this reaction, we monitored the solution using time-dependent HPLC analysis. However, the PPC signal could not be detected in the HPLC column due to the presence of positively charged trimethylammonium groups, which hinder the passage of PPC through C_{18}-modified beads. Therefore, to calculate the percentage of the hydrolyzed product, we measured the relative absorbance of 0.5 mM 7-(diethylamino)-4-(hydroxymethyl)-2H-chromen-2-one using HPLC, which served as a reference representing 100% hydrolysis. Based on this reference, the percentage of PPC hydrolyzed at different time points was calculated.

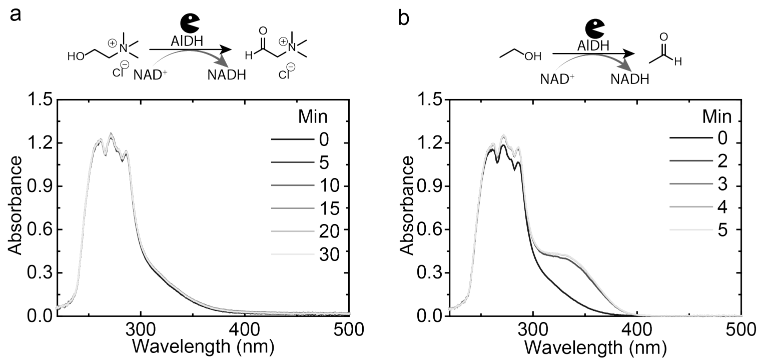

Figure S7. Substrate specificity of AlDH towards choline chloride and ethanol. Time-dependent absorption spectra demonstrating (a) no conversion of NAD^+ to NADH in the presence of choline chloride and (b) conversion of NAD^+ to NADH in the presence of ethanol. ([NAD^+] = 0.1 mM, [Choline] = [Ethanol] = 0.1 mM, [AlDH] = 3.3 mU/μL).

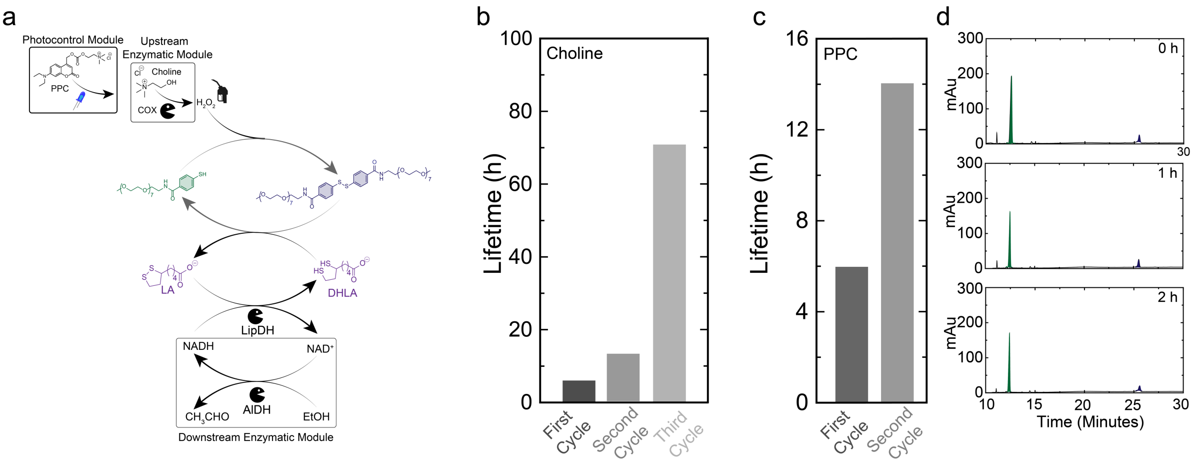

Figure S8. (a) Schematic showing photoinitiated redox ERN with downstream and upstream enzymatic modules for multiple refueling of the system. Lifetime of each cycle when fueled using (b) 0.2 mM choline and (c) 0.3 mM PPC followed by light irradiation with 5 mW/cm^2 420 nm LED for 30 minutes. (d) Time dependent HPLC chromatogram of redox ERN containing upstream and downstream enzymatic modules when fueled with choline in presence of higher concentration of LiPDH. (Conditions for (b) and (c): [**A**] = 0.1 mM, [COX] = 1.3 mU/μL, [LipDH] = 5 mU/μL, [AlDH] = 6 mU/μl, [NADH] = 0.25 mM, [DHLA] = 0.25 mM, [EtOH] = 100 mM. Conditions for (d) is same except [LipDH] = 8.3 mU/μL).

Note: To investigate whether a higher concentration of LipDH will lead to more number of transient cycles, we increased the [LipDH] from 5 mU/μL to 8.3 mU/μL. However, we observe no disulfide formation during the first cycle of fueling which is due to increase in [LipDH], thus leading to an excessive increase in the rate of the reducing half of the ERN.

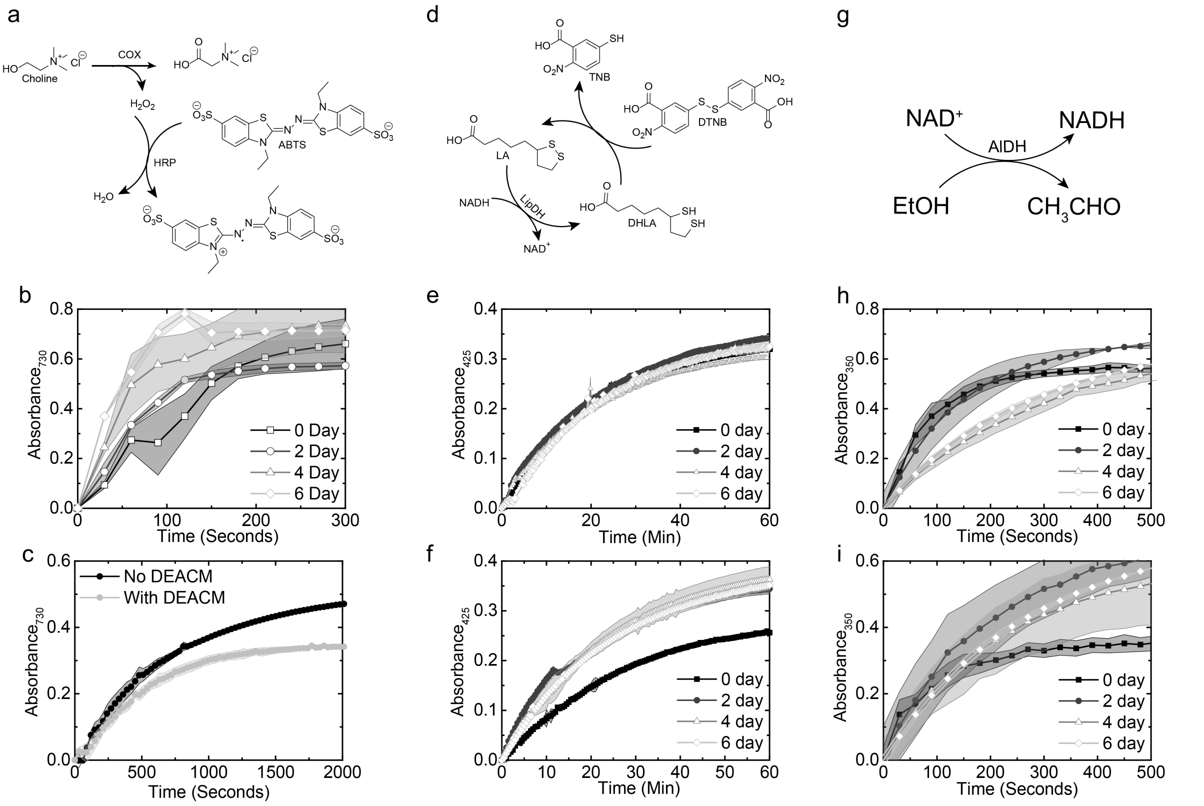

Figure S9. Ageing and stability studies of enzymes in presence and absence of DEACM to decipher the reason of increased lifetime in subsequent refueling cycles and failure of third cycle while refueling using PPC. (a) Schematic showing oxidation of ABTS using HRP in presence of COX-generated H_2O_2 which was utilized to monitor the enzyme kinetics of COX. (b) Enzymatic activity of COX at different time points ([ABTS] = 0.3 mM, [HRP] = 2 mU/μL, [Choline] = 0.3 mM, [COX] = 0.6 mU/μL) and (c) enzymatic activity of COX in presence and absence of DEACM ([ABTS] = 0.3 mM, [HRP] = 2 mU/μL, [Choline] = 0.3 mM, [COX] = 0.5 mU/μL, [DEACM] = 0.5 mM). (d) Schematic showing reduction of DTNB to TNB using enzymatically generated DHLA by LipDH, which was used to monitor the time dependent enzymatic activity and stability of LipDH. Corresponding activity of the LipDH over 6 days (e) in absence and (f) in presence of DEACM ([DTNB] = 0.5 mM, [LA] = 0.5 mM, [NADH] = 1 mM, [DEACM] = 0.5 mM, [LipDH] = 5 mU/μL). (g) Schematic showing conversion of NAD^+ to NADH in presence of ethanol and AlDH and corresponding activity of the AlDH over 6 days (e) in absence and (f) in presence of DEACM ([NAD^+] = 0.5 mM, [EtOH] = 2 mM, [AlDH] = 4 mU/μL). Studies were done in pH = 7.4 PB containing 1 wt% BSA at 25 °C. DTNB, DEACM, ABTS was dissolved in high concentration in DMSO and added into the reaction mixture to keep the DMSO content as low as possible.

Note: To find out the reason of increased lifetime in subsequent refueling cycles and failure of third cycle while fueling using PPC we investigated the time dependent stability of the enzymes in presence and absence of DEACM (the photoprotecting group). Time dependent activity of COX, LipDH, AlDH does not show any significant difference both in presence and absence of DEACM. The plot in Figure S9c shows that the absorbance changes with time slows down over time in presence of DEACM for COX. This is due to competitive oxidation of ABTS and coumarin in presence of HRP and H_2O_2 which leads to lesser oxidation of ABTS when coumarin is present and is not due to enzymatic inhibition.

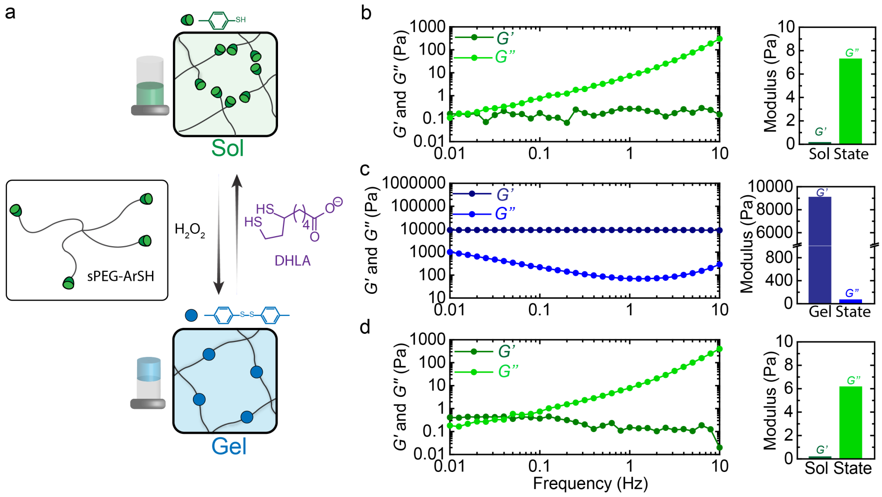

Figure S10. Schematic depicting reversible sol-gel-sol transformation upon H_2O_2 addition to sPEG-ArSH solution and DHLA addition to disulfide crosslinked hydrogel, respectively. Oscillatory frequency sweep rheological measurement of reversible sol-gel-sol transformation of redox responsive hydrogel in (b) initial state before H_2O_2 addition, (c) on hydrogelation using H_2O_2, and (d) sol state again after DHLA addition. The respective right panel shows the moduli at 1 Hz frequency and 1% strain for initial state, hydrogel state, and final state. ([sPEG-ArSH] = 2.5 mM, [H_2O_2] = 15 mM, [DHLA] = 20 mM).

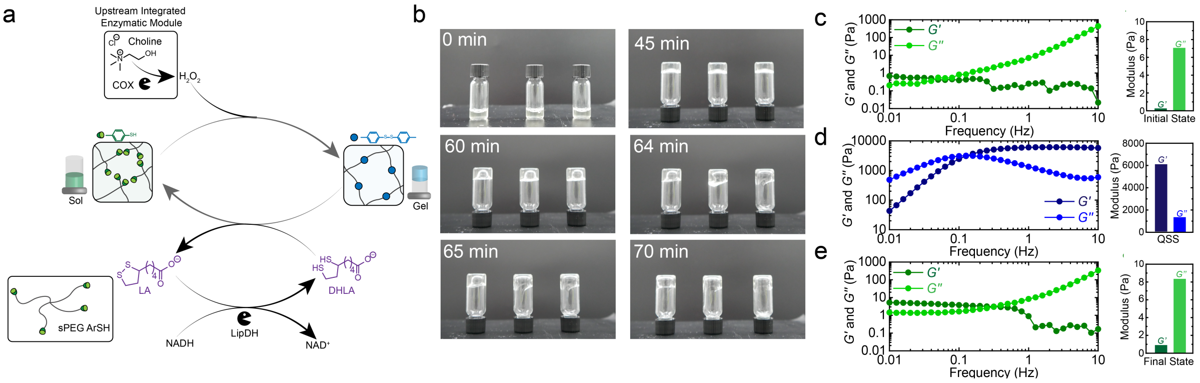

Figure S11. (a) Schematic showing integration of redox ERN containing upstream enzymatic module to sPEG-ArSH for dissipative hydrogel formation. (b) Snapshot series of the dissipative hydrogels fueled by choline with varying LipDH concentration (from right to left: 0.025 U/µL, 0.023 U/µL, 0.021 U/µL of LipDH). Ex-situ rheology data of (c) sol (no choline), (d) QSS (t = 1 h, and (e) final sol state after 12 h. The right-side panel shows the respective moduli in the sol state, QSS, and at the end of the cycle at a frequency of 1 Hz and strain = 1%. ([sPEG-ArSH] = 2.5 mM, [DHLA] = 2.5 mM, [NADH] = 60 mM, [COX] = 0.03 U/µL, [Choline] = 9 mM).

Note: The system shows only a mild dependence on lifetime with the enzyme concentration presented here. A higher enzyme concentration (0.05 U/µL) leads to formation of a very weak gel and a lower enzyme concentration such as 0.01 U/µL does not lead to melting of the gel state. We hypothesize integration of the downstream enzymatic regeneration system will allow us to more finely tune the lifetime and achieve a much broader programmable lifetime however this is currently beyond the scope of present study.

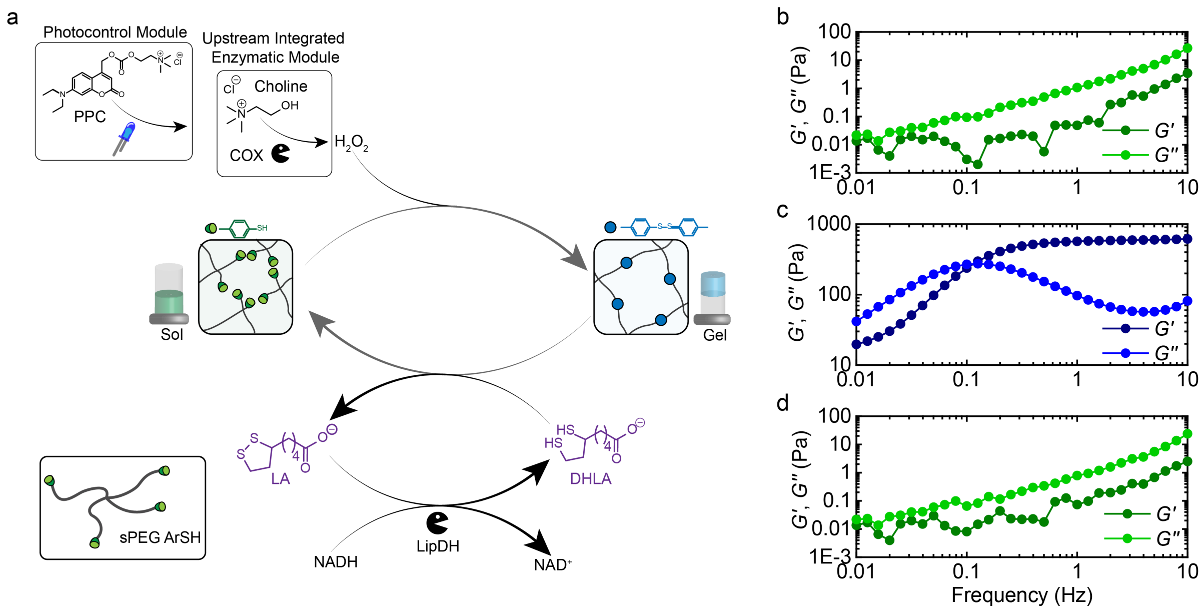

Figure S12. (a) Schematic showing integration and photoactivation of redox ERN containing upstream enzymatic module to sPEG-ArSH for dissipative hydrogel formation. *Ex-situ* rheology data of (b) sol (unirradiated), (c) QSS (t = 1 h, 30 min irradiated using 420 nm LED), and (d) final sol state after 10 h. ([sPEG-ArSH] = 2.5 mM, [DHLA] = 2.5 mM, [NADH] = 60 mM, [LipDH] = 0.025 U/μL, [COX] = 0.03 U/μL, [PPC] = 9 mM).

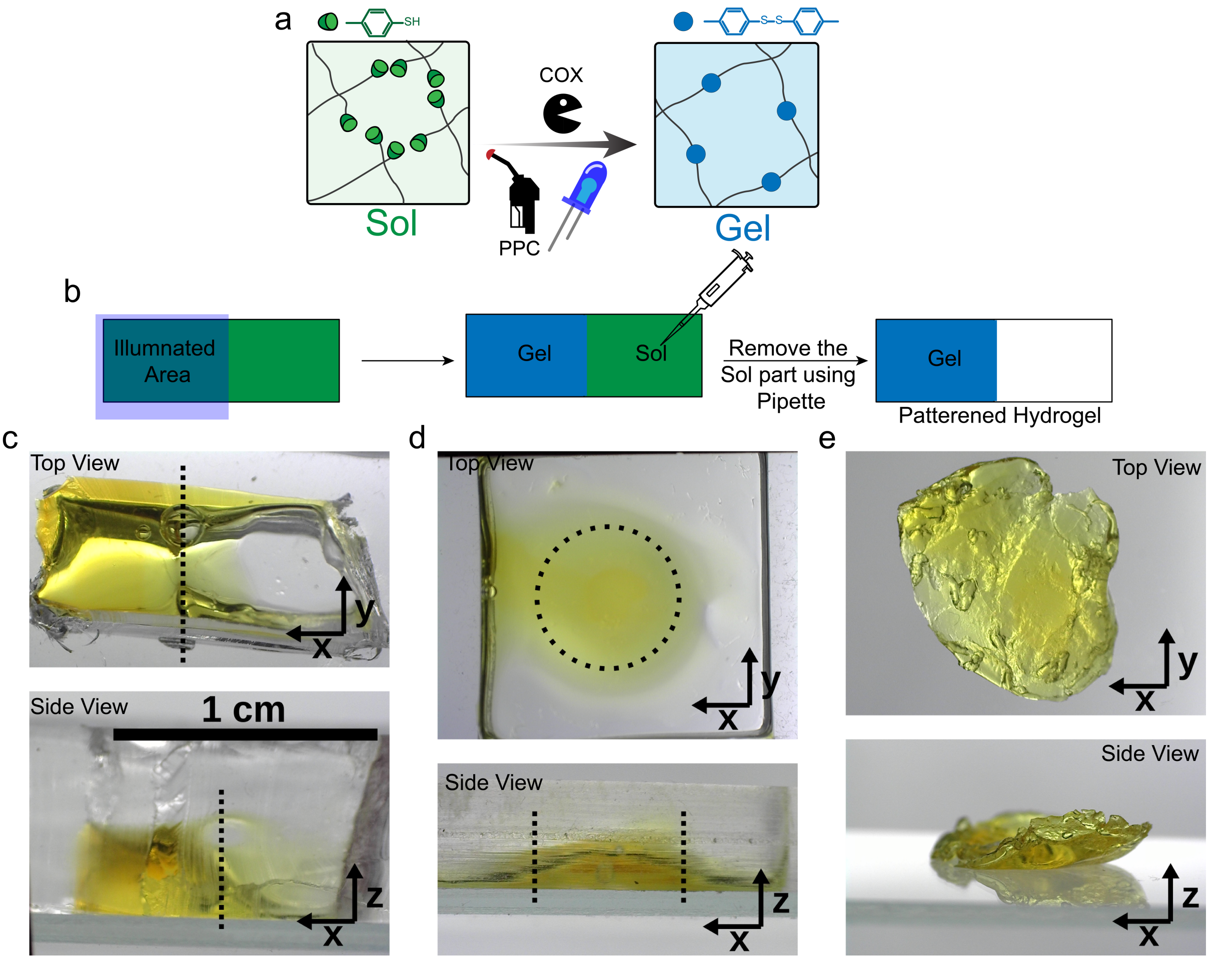

Figure S13. Schematic showing (a) hydrogel formation using sPEG-ArSH in presence of COX and PPC driven by disulfide crosslink formation when PPC is photo-released using 420 nm LED and (b) photo patterned hydrogel formation using spatial irradiation with a photomask. The irradiated area forms a hydrogel due to photo-release of choline and the masked area remains as a sol which could be removed to develop the gel. Respective micrographs of the patterned hydrogel when (c) half of the solution was irradiated and (d) a circular area was irradiated using 10 mW/cm^2 420 nm LED and the gel was developed. The dotted line shows the irradiated area using a photomask. (e) Micrographs of the gel after taking it out of the sample chamber shows the robustness of the patterned hydrogel. ([sPEG-ArSH] = 2.5 mM, [COX] = 0.03 U/μl, [PPC] = 9 mM).

B) Characterization spectra

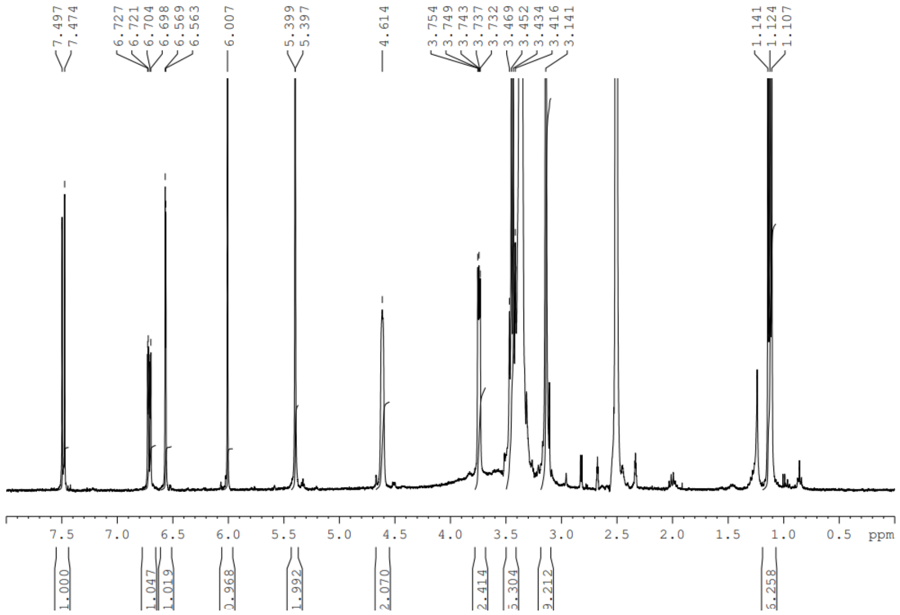

Figure S14. ^{1}H NMR of **PPC (6)** DMSO-d_6, 400 MHz, 298 K.

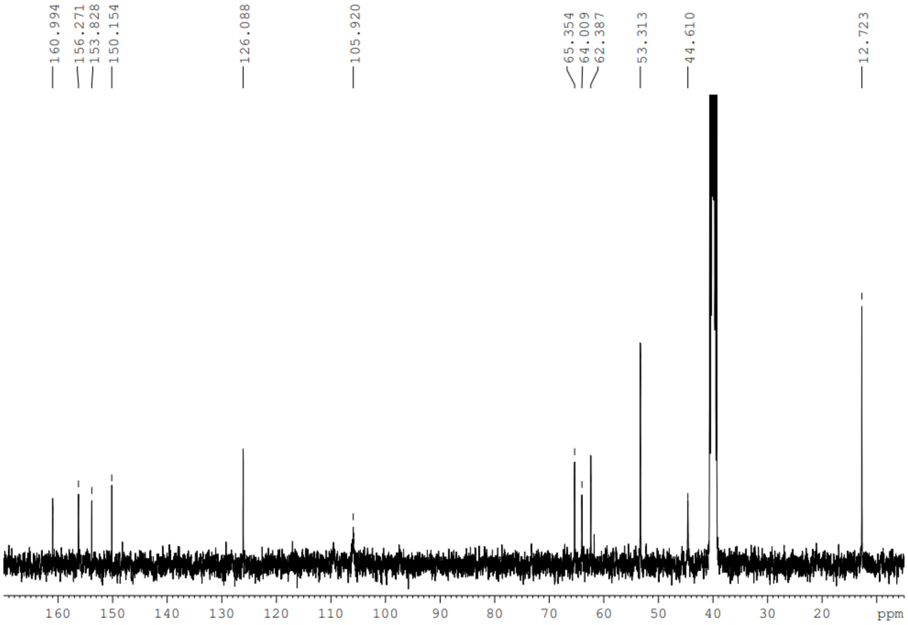

Figure S15. ^{13} NMR of **PPC (6)** DMSO-d_6, 100 MHz, 298 K.

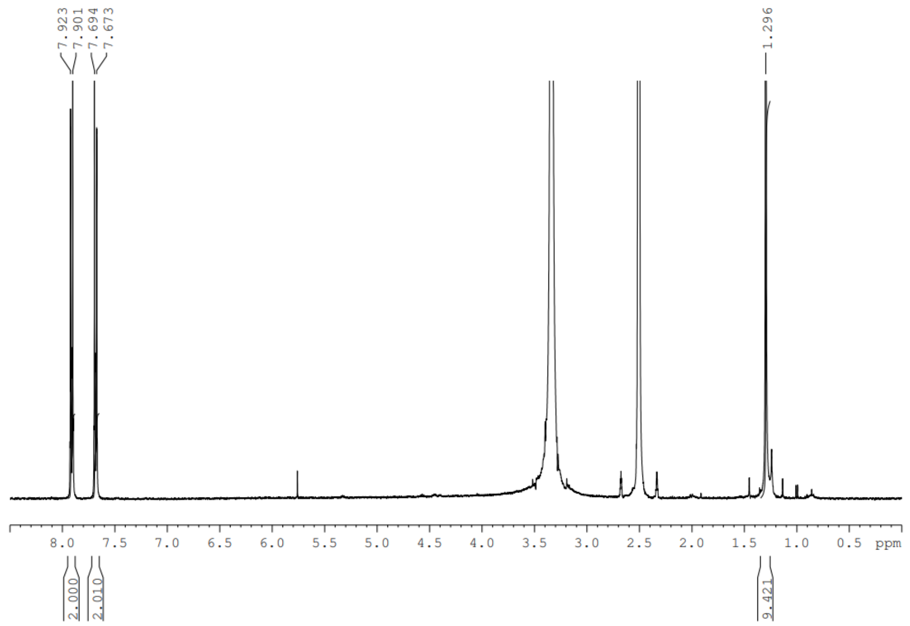

Figure S16. ^{1}H NMR of **9** DMSO-d_6, 400 MHz, 298 K.

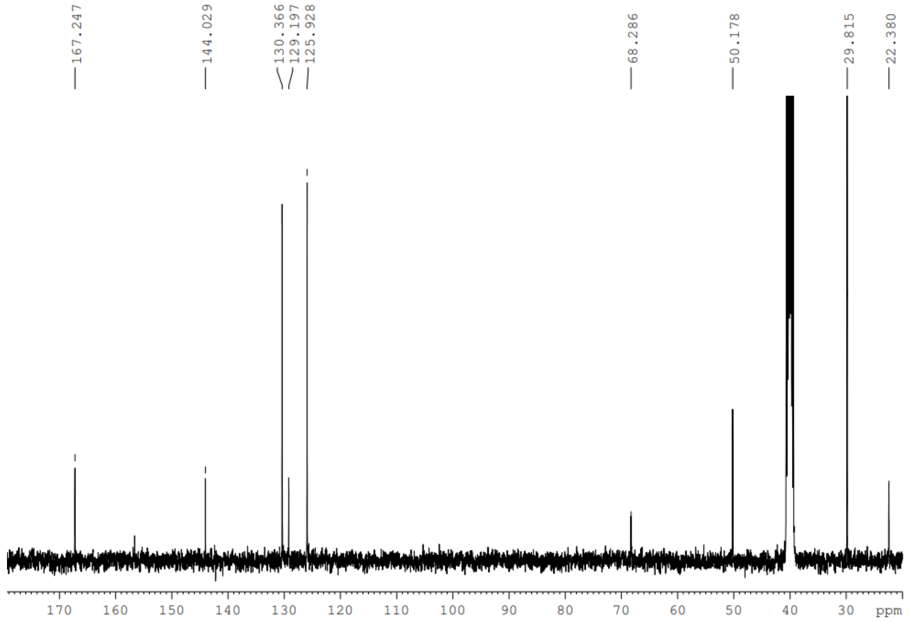

Figure S17. ^{13}C NMR of **9**. $CDCl_3$, 100 MHz, 298 K.

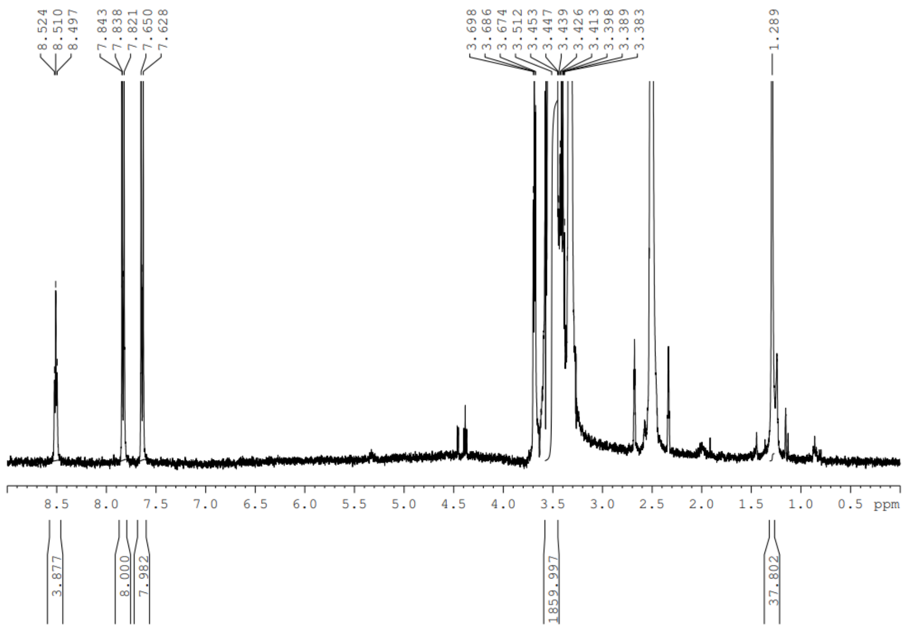

Figure S18. ^{1}H NMR of **sPEG-Ar-S-Strt (11)**. DMSO-d_6, 400 MHz, 298 K.

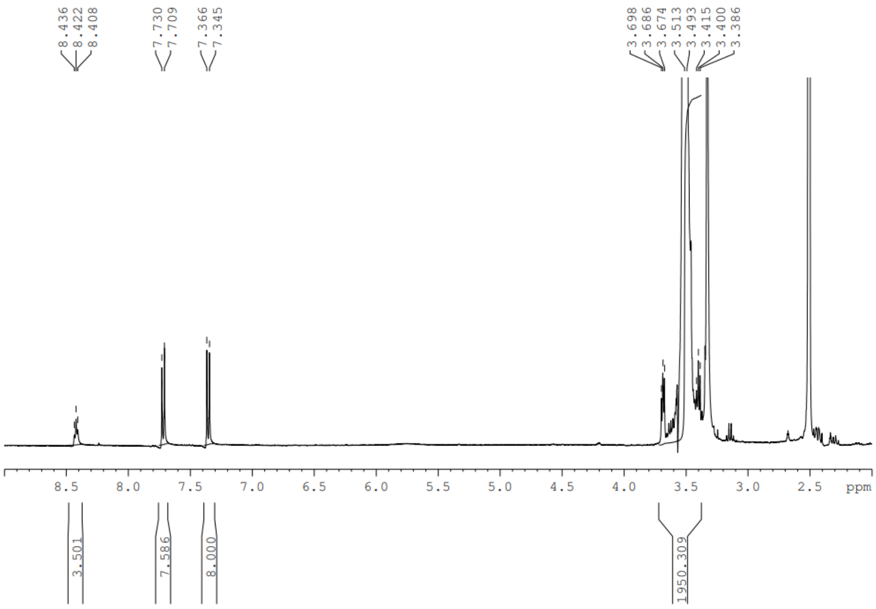

Figure S19. ^{1}H NMR of **sPEG-ArSH (12)**. DMSO-d_6, 400 MHz, 298 K.

C) References

[S1] Allan, D. B., Caswell, T., Keim, N. C., van der Wel, C. M., & Verweij, R. W. (2024). soft-matter/trackpy: v0.6.4 (v0.6.4). Zenodo. https://doi.org/10.5281/zenodo.12708864.
